# Unfavorable food consumption in children up to school entry age: results from the nationwide German KiESEL study

**DOI:** 10.3389/fnut.2024.1335934

**Published:** 2024-07-01

**Authors:** Clarissa Spiegler, Sara Jansen, Leonie Burgard, Friederike Wittig, Anna-Kristin Brettschneider, Andrea Schlune, Thorsten Heuer, Andrea Straßburg, Silvia Roser, Stefan Storcksdieck Genannt Bonsmann, Regina Ensenauer

**Affiliations:** ^1^Department of Nutritional Behaviour, Max Rubner-Institut (MRI) – Federal Research Institute of Nutrition and Food, Karlsruhe, Germany; ^2^Department of Child Nutrition, Max Rubner-Institut (MRI) – Federal Research Institute of Nutrition and Food, Karlsruhe, Germany; ^3^Max Rubner-Institut (MRI) – Federal Research Institute of Nutrition and Food, Presidential Office, Karlsruhe, Germany

**Keywords:** infants, young children, toddlers, preschoolers, food consumption, food-based dietary guidelines, National Nutrition Survey

## Abstract

**Introduction:**

Evidence points toward the early life being crucial for preventing nutrition-related diseases. As promotion of healthier food preferences in toddlerhood and preschool age might still modulate the trajectories of disease risk, understanding diet in these age groups is necessary. The objective was to analyze food consumption and diet quality of 1–5-year-old children living in Germany in relation to age and sex.

**Methods:**

Data from 890 children, a subsample of the representative, cross-sectional Children’s Nutrition Survey to Record Food Consumption (KiESEL) conducted by the German Federal Institute for Risk Assessment in 2014–2017, were analyzed. Dietary data were collected using food records (3 consecutive plus 1 independent day). Diet quality was evaluated against the German food-based dietary guidelines (FBDG).

**Results:**

Consumption of unfavorable foods (e.g., sweets, soft drinks) exceeded the recommended maximum of 10% of energy intake (E%) by a multiple in all age and sex groups (medians: 24.8–35.8 E%). Preschoolers consumed more unfavorable foods than toddlers and boys more than girls. More than half of the children exceeded the recommendation for meat intake (medians: 2.3–3.2% of the total food consumption (%TFC) vs. 2 %TFC), especially preschoolers. In nearly all children, vegetable consumption was too low (medians: 4.2–4.5 %TFC vs. 12 %TFC). Also, milk/milk product consumption was below recommendations, more so in preschoolers (median: 12.0 %TFC ♂, 11.9 %TFC ♀ vs. 18 %TFC) than in toddlers (median: 16.1 %TFC ♂, 19.6 %TFC ♀). In toddlers and preschoolers with overweight or obesity, adherence to dietary recommendations of these food groups was largely similar to that of the total sample. Overall, 5-year-olds showed an unhealthier dietary pattern than 1-year-olds, which already emerged at age 2 years and became more evident at age 3 years.

**Discussion:**

An adverse dietary pattern arises and even deteriorates at a very young age, showing sex-specific aspects. High attention from public health and research needs to be given to toddlerhood and even earlier life phases, e.g., to develop more age-specific FBDGs, aiming at reducing unhealthy food consumption.

## 1 Introduction

The high prevalence of overweight and obesity in children and adolescents worldwide (1), together with the risk of its progression into adulthood and association with adverse health outcomes such as cardiovascular diseases and type 2 diabetes mellitus (T2DM) later in life (2) requires actions. One modifiable risk factor for childhood overweight and related diseases is diet (3, 4). In order to address the prevention of nutrition-related diseases, actions ought to focus on the early years of life for two reasons: Firstly, the underlying subclinical conditions of nutrition-related diseases, e.g., fatty streaks and fibrous plaques in atherosclerosis or insulin resistance in T2DM, start to manifest early in life (3, 5). Metabolic alterations acquired early in life are likely to progress if risk factors persist (3). Secondly, some studies suggest that dietary patterns, once established, remain relatively stable throughout childhood and adolescence (6, 7). At the same time, it seems that during preschool age, development of eating behavior remains shapeable and healthier food preferences can be attained (8) to potentially disrupt further disease progression. Therefore, the early years of life including preschool age are vital when it comes to designing public health measures for the prevention of nutrition-related diseases. Nutrition surveys assessing the dietary intake of the various young age groups within this early phase of life as well as addressing sex-specific differences in food consumption are scarce and therefore much needed.

In order to develop preventive concepts specific to children within the first years of life, such as tailored nutritional recommendations, it is important to differentially study their diet in relation to relevant factors such as age. Therefore, this analysis explores the food consumption of children aged ≥12 months to ≤5 years (hereafter referred to as 1–5 years of age) living in Germany according to age and sex. Diet quality is investigated in relation to the current German food-based dietary guidelines (FBDG) for children and adolescents (9), including analysis of a subgroup with overweight or obesity, and differences in food consumption throughout the first years of life up to school entry age are analyzed by comparing age groups year by year.

## 2 Materials and methods

### 2.1 Data assessment

The representative, cross-sectional Children’s Nutrition Survey to Record Food Consumption (*Kinder-Ernährungsstudie zur Erfassung des Lebensmittelverzehrs*, KiESEL) was conducted from 2014 to 2017 by the German Federal Institute for Risk Assessment (*Bundesinstitut für Risikobewertung*, BfR) as a module of the German Health Interview and Examination Survey for Children and Adolescents Wave 2 (*Studie zur Gesundheit von Kindern und Jugendlichen in Deutschland Welle 2,* KiGGS Wave 2). KiGGS is part of the national health monitoring performed by the German Robert Koch-Institute (RKI) (10, 11). Within KiESEL, food consumption data of children from ≥6 months to ≤5 years of age living in Germany were collected. Details on the sampling methods are described elsewhere (10). In brief, the KiESEL participants were randomly drawn from the cross-sectional KiGGS Wave 2 sample, with 167 sample points across Germany, targeting a sample size of at least *n* = 1,000 (10). The study was approved by the ethics committee of the Berlin Chamber of Physicians (Eth-28/13) and the German Federal Commissioner for Data Protection and Freedom of Information. The primary caregivers of each child enrolled in the study gave written informed consent. The STROBE-nut checklist was used when writing this report (12) (Supplementary Table S1).

The total KiESEL sample includes *n* = 1,104 children. For *n* = 96 children of those, no weighed food record data were available (10). Infants (≥6 months to <12 months, *n* = 118) will be reported separately. For the present analysis, a subsample of children aged 1–5 years with available food record data (*n* = 890) was used (Supplementary Figure S1). Age specifications refer to completed years of life at the beginning of data collection, i.e., “1 year” includes all children aged 1.0–1.9 years. Due to the time lag between recruitment and data collection, the sample includes *n* = 62 (6.2%) children aged 6 years when data were collected. Two age groups were defined according to the German FBDG for children and adolescents aged 1–18 years, the so-called Optimized Mixed Diet (OMD) recommendations (9). The younger group includes children from ≥1.0 to ≤3.9 years (hereafter referred to as “aged 1–3 years” or “toddlers”) and the older group includes children ≥4.0 years (hereafter referred to as “aged 4–5 years” or “preschoolers”; including those aged 6 years). The KiESEL study design and survey protocol have been published previously (10, 13).

Food consumption was assessed using a parent-administered weighed food record on three consecutive days and an additional independent fourth day, scheduled 2–16 weeks later (3 + 1 design). During a home visit prior to food recording, parents received instructions by trained nutritionists and were equipped with kitchen scales and a journal with pre-printed log pages. The information requested in the journal included details of the food and beverages consumed (e.g., type of preparation, brand) as well as place and time of consumption. For child day care facilities and other out-of-home eating occasions, quantities were estimated with the help of package labels, household measures, or the specifically developed, pilot-tested KiESEL picture book. In cases of ambiguity in protocol entries, parents were called to seek clarification (13).

Body height and weight were measured by trained staff at the time of the home visit. Generally, children only wore underwear when being weighed. In certain cases, a predefined weight was subtracted for clothes or, in small children, for empty nappies (10). Depending on the individual child’s age, age-and sex-specific body mass index (BMI) z-scores were calculated following the protocols of the World Health Organization (WHO) Child Growth Standards for children below 5 years of age (14) or the WHO growth reference for school-aged children and adolescents (15). Children aged 60 months or younger were classified as having overweight or obesity if their BMI z-score was >2 to ≤3 SD or >3 SD, respectively. Children aged 61 months and older were classified as having overweight or obesity if their BMI z-score was >1 to ≤2 SD or >2 SD, respectively (16). Children with BMI z-scores < −2 SD were classified as having underweight. Data on region of residence and socioeconomic status (SES) were collected within KiGGS Wave 2. SES was calculated based on the parents’ education, occupational status, and income (equally weighted) and categorized (low, medium, high) based on quintiles determined in KiGGS Wave 2 (17).

### 2.2 Human milk estimates

Human milk quantities were not reported consistently. If human milk feeding was recorded without quantification, amounts were estimated according to age and feeding frequency (94.5% of cases). Per feed, the estimated amount was 89 mL for children aged 12–17 months and 59 mL for children ≥18 months, as described by Briefel et al. (18). As this method led to daily amounts of human milk considered plausible, no upper threshold was defined. For one participant, human milk feeding was reported as “throughout the day,” without information on feeding frequency and quantity on three of the four protocol days. On day four, consumption of human milk was quantified (200 mL), and thus, 200 mL/day were used as an estimate for each of the protocol days. Amounts of other drinking milk (e.g., cow’s milk) were subtracted (19).

### 2.3 Food groups

Each recorded food item was assigned to one food group. As a template for food groups, the OMD recommendations (9) were used, and the additional food group of nuts was added (Supplementary Table S2). Last revised in 2017, the OMD recommendations list age group-specific amounts for daily or weekly consumption of 11 food groups and their suggested share of total food consumption (%TFC; TFC = sum of consumed amounts (g/day) of all OMD recommended food groups). These 11 food groups are categorized as either “eat plenty,” “eat in moderation,” “eat sparingly” (all three categories summarized as “recommended”) or “tolerated” (e.g., sweets, soft drinks, or sugary breakfast cereals, Supplementary Table S2); hereafter referred to as “unfavorable foods.” The quantities of recommended food groups are calculated to provide 90% of a child’s daily energy requirements, and 10% of energy intake (E%) remain to accommodate energy intake from unfavorable foods (9). Energy requirements are based on the assumption of a low physical activity level of 1.4 (9).

The quantities of the recommended food groups aim to result in nutrient intakes in line with the current dietary reference values of Germany, Austria and Switzerland, considering fat and saturated fatty acids, eight vitamins (retinol equivalents, α-tocopherol equivalents, vitamin C, thiamin, riboflavin, pyridoxine, folate, vitamin D), and six minerals (calcium, phosphorus, magnesium, iron, zinc, iodine) (9). It should be noted that reference values for vitamin D and iodine are not reached with a diet according to the OMD recommendations. The authors conclude that supplementation or fortification is necessary to ensure adequate intake of these two critical nutrients (9).

For the present analysis, OMD food groups were slightly modified. In the OMD recommendations, plain nuts and seeds are not listed as a separate food group (9). To quantify the consumption of plain nuts and seeds as a valuable and recommended source of nutrients (20), they are presented separately in the current analysis (Supplementary Table S2), while salted nuts were considered as unfavorable foods. The two groups of “bread and cereals” and “starchy side dishes” serve as mutual substitutes and therefore were combined into the group of “carbohydrate foods.” Since the OMD recommendations for fish and eggs are given as weekly instead of daily amounts, these figures were divided by 7 to facilitate comparison with the daily recommendations for the other food groups.

In the OMD recommendations, milk and milk products are displayed as “milk equivalents” (9), which are roughly computed based on the protein content of the different milk products in relation to plain cow’s milk (informal information: Research Department of Child Nutrition, University Hospital of Pediatrics and Adolescent Medicine, Ruhr-University Bochum, 2022). For comparison with the OMD recommendations, milk equivalents were calculated accordingly in KiESEL for the different subgroups of milk products by multiplying the consumed amount per subgroup by a factor. This milk equivalent factor was derived as ratio of protein content of the subgroup to the protein content of raw milk (in g per 100 g raw milk) as listed in the German Nutrient Database (*Bundeslebensmittelschlüssel*, version 3.02) (21). The so-derived milk equivalent factors as well as the equation are displayed in Supplementary Figure S2. In the following, the term “milk and milk products” refers to the calculated milk equivalents.

The components of most of the composite dishes (e.g., pizza, casseroles) were disaggregated and classified individually. The disaggregation of composite dishes was based on the information of recipes provided by the parents or the recipes available in the German Nutrient Database (21). Exemptions were made for sweet foods and dishes such as cake, sweet semolina pudding, and sweet pancakes, as well as for bread, bread rolls, pasta, potato products, and commercial complementary foods (CCF), which were assigned as a whole. Foods such as infant and follow-on formula or granulated herb-or fruit-based teas were considered as ready-to-drink products, which included the weight of the liquid used for preparation.

### 2.4 Misreporting

Misreporting of energy intake was identified following the protocol by the European Food Safety Authority (EFSA) (22) using the Goldberg cut-off method updated by Black (23). A physical activity level of 1.4 and 1.6 was assigned to children aged 1–3 years and 4–5 years, respectively, in order to calculate cut-offs (Supplementary Table S3) (22). Following the recommendations by EFSA, Schofield equations were used to calculate the basal metabolic rate, and under-and over-reporters were not excluded to avoid bias (22).

### 2.5 Statistics

**Figure 1:**
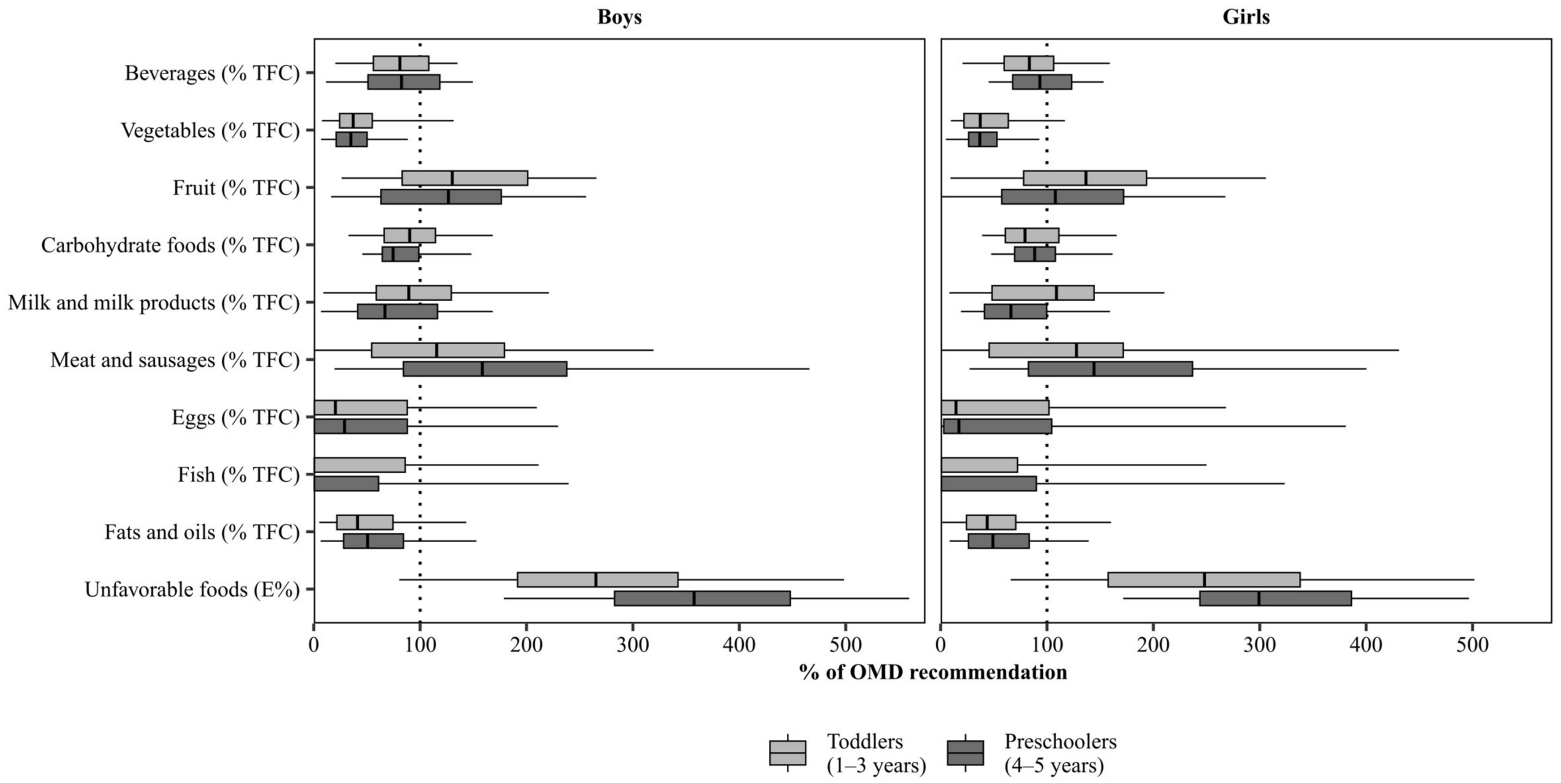
Food consumption in KiESEL toddlers and preschoolers, stratified by sex. Shown are weighted data. Boxes represent 25th to 75th percentile with the median shown as the vertical line and whisker ends representing the 5th and 95th percentile; outliers not shown. The dotted line represents 100% of the respective OMD recommendation (given in % TFC or E%). %TFC, percentage of total food consumption; E%, percent of energy intake; KiESEL, Children’s Nutrition Survey to Record Food Consumption; OMD, Optimized Mixed Diet.

Measures of the sample’s daily food consumption were calculated from individual values, which are computed as arithmetic mean of all protocol days per child (including non-consumers). The relative contribution of the different food groups to total food consumption was calculated as %TFC. The %TFC was compared with the proportions given by the OMD recommendations. For graphic presentation (Figure 1), each individual %TFC value was calculated in relation to the respective TFC value of the OMD recommendations (Supplementary Table S2), which was set at 100%. For comparison of food consumption by year of age, consumed amounts (g/day) were standardized to 1,000 kcal (i.e., g/1000 kcal) to compensate for age-and sex-related differences in energy intake.

Data were weighted for sex, age, region, regional structure, and household education level to compensate for deviations from the German population of toddlers and preschoolers. For this purpose, a weighting factor designed by the RKI for the total KiESEL sample (0.5–5 years) and based on data reported by the Federal Statistical Office of Germany [Microcensus 2015; for household educational level: Microcensus 2013 (24)] was used. Unweighted data, e.g. for the subgroup of children with overweight/obesity, are marked as such. For all statistical analyses, SAS version 9.4 (SAS Institute, Inc., Cary, NC, United States) was used. Statistical measures include arithmetic mean, standard deviation (SD), 95% confidence interval (CI) of the mean, median, 5th and 95th percentiles (hereafter referred to as P5 and P95, respectively), and standard error of the mean (for g/1,000 kcal only). Although food consumption data were not normally distributed, we decided to primarily display mean values and CIs of the mean to approach average consumption in the population. Median values alone were not considered sufficient as they were zero for some of the food groups. P5 and P95 were chosen to capture extreme ends of the distribution. Differences between two groups were considered significant if the CIs did not overlap, based on three decimals. Differences between more than two groups were tested using the Kruskal-Wallis test and the Dwass-Steel-Critchlow-Flinger method for post-hoc testing (pairwise comparison of all age group combinations, α per food group = 0.05).

## 3 Results

### 3.1 Study characteristics

Table 1 shows characteristics of the KiESEL sample of children aged 1–5 years. Combined prevalences of overweight and obesity were 7.1–16.9%, depending on age group and sex. Less than 15% of all children came from families with a low SES. Under-and over-reporting of energy intake was identified in 5.6 and 1.1% of all children, respectively, according to calculated cut-offs (Supplementary Table S3).

**Table 1:** Characteristics of KiESEL toddlers and preschoolers, stratified by sex.^a^

	Toddlers (1–3 years)		Preschoolers (4–5 years)	
	Boys	Girls	Boys	Girls
*n* (%)	254 (28.4)	247 (27.0)	200 (23.0)	189 (21.5)
Age (years, median)	2.5	2.5	5.3	5.1
**Anthropometric measurements (mean ± SD)**				
Weight (kg)	13.6 ± 2.6	13.2 ± 2.8	19.8 ± 3.0	19.7 ± 3.8
Height (cm)	90.6 ± 8.9	89.9 ± 9.3	112.1 ± 6.7	111.2 ± 7.1
BMI (kg/m^2^)	16.5 ± 1.5	16.2 ± 1.6	15.7 ± 1.4	15.8 ± 1.9
**Weight classification**^b^ **(*n*, %)**				
Underweight	1 (0.5)	5 (1.2)	2 (0.4)	0 (0)
Overweight	13 (6.5)	14 (9.1)	15 (8.8)	21 (11.8)
Obese	1 (0.6)	1 (0.4)	4 (3.1)	3 (5.1)
**Socioeconomic status**^c^ **(*n*, %)**				
Low	14 (11.9)	15 (13.8)	13 (16.4)	11 (10.4)
Medium	145 (62.3)	150 (62.7)	117 (60.0)	123 (72.1)
High	95 (25.9)	82 (23.5)	68 (23.6)	54 (17.5)
**Residential area**^d^ **(*n*, %)**				
North	37 (16.7)	28 (15.5)	23 (15.3)	23 (17.2)
East	86 (21.2)	95 (20.0)	66 (18.1)	53 (16.8)
South	79 (27.8)	59 (30.4)	65 (29.7)	54 (29.0)
West	52 (34.3)	65 (34.1)	46 (36.9)	59 (37.0)

a Weighted data (n unweighted). Small deviations from 100.0% can occur due to rounding. BMI, body mass index; KiESEL, Children’s Nutrition Survey to Record Food Consumption.

b BMI categories were defined as follows: children ≤ 60 months: BMI z-score < −2 SD underweight, >2 to ≤3 SD overweight, >3 SD obesity; children ≥ 61 months: BMI z-score < −2 SD underweight, >1 to ≤2 SD overweight, >2 SD obesity (16).

c Data on SES were missing for *n* = 3 children.

d Federal states of Germany were assigned as follows: North: Schleswig-Holstein, Hamburg, Lower Saxony, Bremen; East: Berlin, Brandenburg, Mecklenburg-Western Pomerania, Saxony, Saxony-Anhalt, Thuringia; South: Baden-Wuerttemberg, Bavaria; West: North Rhine-Westphalia, Hessia, Rhineland-Palatinate, Saarland.

### 3.2 Daily food consumption according to age and sex

Mean energy intake (kcal/day) and food consumption (g/day) of toddlers and preschoolers stratified by sex are shown in Table 2. Values for median, P5, and P95 are presented in Supplementary Table S4. In all age and sex groups, beverages, milk and milk products, unfavorable foods, fruit, and carbohydrate foods were the food groups consumed in the largest amounts. Among unfavorable foods, sweets, soft drinks, and sweetened milk products were the largest subgroups. Within the group of total fruit, fruit juice and smoothies made up one third to half of the mean total fruit consumption. Fish, eggs, fats and oils, as well as nuts were consumed in small amounts (≤10 g/day).

**Table 2:** Energy intake and food consumption in KiESEL toddlers and preschoolers (in kcal/day or g/day), stratified by sex (mean, 95% CI of the mean).^a^

	Toddlers (1–3 years)				Preschoolers (4–5 years)			
	Boys *n* = 254		Girls *n* = 247		Boys *n* = 200		Girls *n* = 189	
	Mean (SD)	95% CI (mean)	Mean (SD)	95% CI (mean)	Mean (SD)	95% CI (mean)	Mean (SD)	95% CI (mean)
Total energy intake (kcal/day)	**1,041** (231)	1,013–1,069	**982** (245)	951–1,012	**1,378** (224)*	1,348–1,408	**1,230** (236)*	1,197–1,263
**Food groups (g/day)**								
Beverages	398 (241)	368–427	416 (273)	382–450	552 (334)*	507–597	598 (366)*	547–649
Vegetables	69 (51)	63–75	71 (58)	64–79	76 (55)	69–84	77 (49)	70–84
Fruit (total)^b^	197 (118)	183–211	198 (145)	180–216	232 (151)*	211–252	208 (159)*	185–230
Fruit juice and smoothies	67 (86)	56–77	74 (112)	60–87	112 (124)	95–129	87 (124)	70–105
Nuts	1 (4)	0–1	1 (4)	1–1	1 (3)	0–1	1 (3)	1–2
Carbohydrate foods (total)	153 (70)	144–161	141 (70)	133–150	177 (63)*	169–186	186 (63)*	177–195
Bread and cereals	80 (51)	74–86	74 (59)	67–82	87 (45)	81–93	87 (38)*	82–92
Starchy side dishes	73 (42)	68–78	67 (40)	62–72	90 (51)*	83–97	99 (58)*	91–107
Milk and milk products^c^	232 (165)	212–252	231 (160)	212–251	234 (164)	212–256	206 (123)	189–223
Meat and sausages	34 (28)	31–38	34 (28)	30–37	56 (37)*	51–61	51 (33)*	46–56
Eggs	8 (11)	6–9	9 (14)	7–10	9 (12)	8–11	10 (15)	8–12
Fish	7 (11)	5–8	6 (10)	4–7	8 (17)	5–10	9 (16)*	7–11
Fats and oils	7 (6)	6–8	7 (6)	6–8	10 (8)*	9–11	9 (6)*	8–10
Unfavorable foods (total)^b^	**197** (197)	173–221	**155** (136)	138–172	**310** (206)*	282–338	**228** (166)*	205–251
Sweets	69 (48)	63–75	61 (44)	55–66	**112** (58)*	105–120	**93** (60)*	84–101
Soft drinks	74 (181)	52–95	48 (112)	34–62	**118** (163)*	96–140	**76** (138)	57–95
Sweetened milk products	41 (50)	35–47	36 (47)	31–42	56 (88)	44–68	46 (59)	37–54
**Combined food groups (g/day)**								
Beverages + soft drinks + juices + smoothies	538 (298)	502–574	537 (299)	500–575	783 (313)*	740–825	761 (358)*	711–811
Milk and milk products + sweetened milk products^d^	285 (167)	265–305	279 (165)	258–299	307 (182)	282–331	265 (129)	247–283

a Weighted data (n unweighted). CI, confidence interval; KiESEL, Children’s Nutrition Survey to Record Food Consumption; SD, standard deviation.

b Note that only selected subgroups are presented. Hence, the sum of subgroups does not correspond to the amount of the superordinate group.

c As milk equivalents.

d With sweetened milk products calculated as milk equivalents.

*Significant difference between preschoolers and toddlers of the same sex; bold = significant difference between boys and girls within one age group (non-overlapping 95% CIs).

*Age-specific differences* were observed for the majority of the food groups: In both sexes, preschoolers consumed more beverages, total carbohydrate foods, starchy side dishes, meat and sausages, fats and oils, total unfavorable foods, and sweets than did toddlers. Some age-related differences were observed in one sex only: In boys, total fruit, fruit juice and smoothies, as well as soft drinks were consumed in higher quantities in preschoolers than in toddlers, while in girls, the same age-related difference was seen for bread and cereals as well as fish.

*Sex-specific differences* were identified for unfavorable foods, of which boys consumed more than girls in both age groups.

### 3.3 Comparison of age-, sex-, and BMI-specific food consumption to the OMD recommendations

Figure 1 shows the food consumption (in %TFC or E%) in relation to the respective OMD recommendation that is illustrated by the dotted line at 100%. Numeric values are presented in Supplementary Tables S5, S6, and reference values expressed as %TFC or E% are shown in Supplementary Table S2. In the following, medians and, if relevant, other quantiles such as P5 and P95 are compared to the OMD recommendations to provide an estimate of the share of children meeting the respective recommendation.

In more than half of the children of all age and sex groups, the %TFC of beverages was below the OMD recommendation (range of medians: 30.0–34.5 %TFC, depending on age and sex group, vs. recommended 37 %TFC). If juices and smoothies as well as soft drinks were additionally considered to quantify consumption of liquids (Supplementary Tables S5, S6), median consumption was 39.9–46.9 %TFC, depending on age and sex group. Thus, the recommended quantity but not quality (sugar-free only) for beverages would have been met. While the recommended share for fruit consumption was exceeded by more than half of all children (range of medians: 11.9–15.0 %TFC, depending on age and sex group, vs. recommended 11 %TFC), vegetable consumption fell short of the OMD recommendations: Median consumption reached only about a third of the recommended 12 %TFC (range of medians: 4.2–4.5 %TFC, depending on age and sex group), and in preschoolers particularly, more than 95% did not reach this recommendation (P95: 10.6 %TFC in boys and 11.1 %TFC in girls).

Regarding total carbohydrate foods, median consumption was below the recommended 13 %TFC (range of medians: 9.7–11.7 %TFC, depending on age and sex group). Median consumption of milk and milk products did not reach the OMD recommendation of 18 %TFC in preschoolers, irrespective of sex (median: 12.0 %TFC in boys and 11.9 %TFC in girls). While in toddler girls, median consumption of milk and milk products (median: 19.6 %TFC) met the recommended share of 18 %TFC, in toddler boys it was below (median: 16.1 %TFC). However, if sweetened milk products were considered as milk and milk products in addition (Supplementary Tables S5, S6), more than half of the children in the different age and sex groups would have met the recommended share (range of medians: 18.5–23.2 %TFC), except for preschool girls (median: 16.1 %TFC).

For more than half of the children, consumption of meat and sausages exceeded the recommended share of 2 %TFC. Specifically, in preschoolers, median consumption was approximately 1.5 times the recommended share (median: 3.2 %TFC in boys and 2.9 %TFC in girls). Median consumption of fish, eggs, and fats and oils was below the respective recommended share. In contrast, median consumption of total unfavorable foods exceeded the recommended maximum of 10 E% by a factor of 2.5 in toddlers (median: 26.5 E% in boys and 24.8 E% in girls) and approximately 3–3.5 in preschoolers (median: 35.8 E% in boys and 29.9 E% in girls). Furthermore, more than 95% of preschoolers exceeded the recommended maximum of 10 E% at least by a factor of 1.7 (P5: 17.9 E% in boys and 17.2 E% in girls). Of note, in about 5% of all children, total unfavorable foods contributed ≥50 E% (range of P95: 49.7–56.0 E%, depending on age and sex group).

*In the subgroup of toddlers and preschoolers with overweight or obesity*, adherence to OMD recommendations for vegetables, milk and milk products, meat and sausages as well as unfavorable foods was largely similar to that of the total sample (see median values in Supplementary Table S7 compared to OMD recommendations in Supplementary Table S2). Unlike for the total sample, subgroup comparison was based on unweighted data.

### 3.4 Food consumption across the early life phase

**Figure 2:**
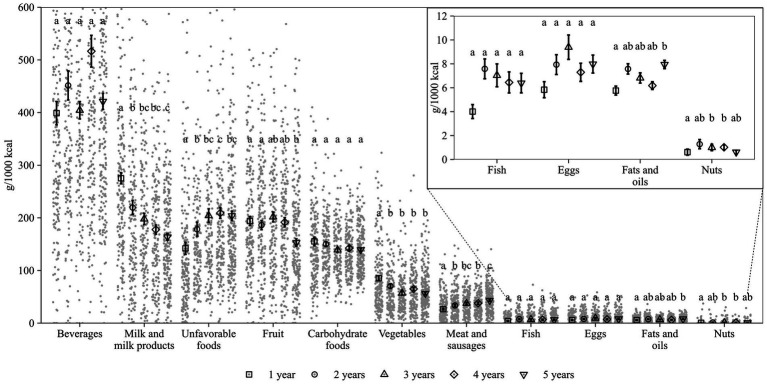
Food consumption in KiESEL toddlers and preschoolers, stratified by age. Shown are weighted data. Dots display individual values (g/1,000 kcal; y-axis limited to 600 g/1,000 kcal). Mean and standard error of the mean are depicted. Shapes represent the different age groups. Different letters indicate significant differences between age groups (tested using Kruskal-Wallis test and Dwass-Steel-Critchlow-Flinger *post hoc* test, α = 0.05). The insert shows mean and standard error of the mean for food groups consumed in low quantities (≤10 g/1000 kcal on average). KiESEL, Children’s Nutrition Survey to Record Food Consumption.

Consumed amounts of the OMD recommended food groups in g/1,000 kcal for 1-, 2-, 3-, 4- and 5-year-olds are shown in Figure 2 and Supplementary Tables S8, S9. The dietary pattern of the youngest age group differed from that of the older children: For milk and milk products and vegetables, mean consumption per 1,000 kcal was higher in 1-year-olds compared to all older age groups. In addition, mean consumption of unfavorable foods and meat and sausages was lower than in all groups of older children.

When specifically looking at the differences between 1- and 5-year-olds, the dietary pattern of 5-year-olds was characterized by lower mean consumption of milk and milk products, fruit, and vegetables and higher mean consumption of unfavorable foods, meat and sausages, and fats and oils. Some features of a diet composition similar to age 5 years already emerged in children aged 2 years based on the consumed amounts of vegetables being lower and unfavorable foods being higher than that of 1-year-olds (Figure 2). The diet composition of 5-year-olds became more evident in children aged 3 years when the consumption of two further food groups was on a similar level to that of 5-year-olds (milk and milk products and meat and sausages). No age-specific differences in food consumption were seen for beverages, carbohydrate foods, fish, and eggs.

## 4 Discussion

These representative data from Germany show an unbalanced diet among most young children up to school entry age when compared to the German FBDG. In virtually all children, there was a large overconsumption of unfavorable foods (such as sweets and soft drinks), which was most pronounced in preschoolers and in boys. Also, meat was consumed more than recommended, particularly at preschool age. In contrast, an underconsumption of vegetables, unsweetened beverages, fish, and, in older children, milk and milk products was found. These disbalances in diet start early in life and appear to progress until school age. Compared to 1-year-olds, 5-year-olds showed an unhealthier dietary pattern that already emerged at age 2 years and became more evident at age 3 years. A similarly composed diet was associated with overweight and a higher risk of obesity later in childhood (25) and, when consumed in adulthood, with an elevated risk for metabolic syndrome and T2DM (26). As dietary behaviors acquired early in life are likely to persist (6), prevention of nutrition-related diseases ought to start early (27, 28).

The excess consumption of **unfavorable foods** such as sweets and soft drinks is one of the most worrying findings of the present analysis. Other European countries mainly report a consumption of these foods below the levels observed in KiESEL (29–33). However, the composition of “unfavorable” food groups is highly heterogenous between surveys, reaching from sugars and sweets only (30, 31, 33) to the inclusion of cakes, sugar-sweetened beverages (SSBs), sweet desserts, sugary breakfast cereals, and sweetened milk products (as in KiESEL). The inclusion of a broad range of such foods might partly explain the observed high consumption of unfavorable foods already in toddlers. Preschool boys, in particular, consumed more unfavorable foods than did girls of the same age. In other European countries, most nutrition surveys covering this young age group do not present data stratified by sex. One exception is the French dietary survey, according to which 4- to 6-year-old boys consumed more SSBs and sweet bakeries than girls of the same age (34), pointing toward a sex-specific pattern comparable to that observed in KiESEL. Even though statistical tests were not reported, it seems that also in the Netherlands, consumption of sweets and cakes follows a sex-specific pattern similar to KiESEL, which was more pronounced in children aged 4–8 years than in 1- to-3-year-olds (29). In the United Kingdom, the same was found for children aged 4–10 years (33). In Denmark, consumption of sugar and sweets was higher in boys aged 6–7 months compared to girls of the same age, but not in toddlers (30). Our analysis suggests sex-specific differences to occur at an early age, which should be addressed in further research.

Unfavorable foods, together with fruit juice, are the main contributors of free sugar intake in KiESEL (own unpublished data). The estimated free sugar intake exceeded the maximum recommendation of 10 E% set by the WHO (35), more so in preschoolers (boys: 18 E%, girls: 17 E%; aged 3–5 years) than in toddlers (boys and girls: 12 E%; aged 1–2 years) (36). This estimation is based on calculating the mono- and disaccharide intake from soft drinks, sweets, fruit juices, cakes, milk and milk products (excluding lactose), breakfast cereals, and spices/seasoning sauces (36). However, it might still underestimate the true intake, as free sugars are contained in some other food groups as well. Similarly, in a regional German longitudinal study, median free sugar intake in 1,312 children aged 3–18 years constantly exceeded the 10 E% by 1.6 to 1.8 times from 1985 until 2016 (37). Unfavorable foods in our analysis also include SSBs. High consumption of SSBs is associated with increased risks for high blood pressure, dyslipidemia, insulin resistance (38), dental caries, particularly in primary dentition (39), and may increase body fat percentage (40).

In a European comparison, consumption of **meat and sausages** in KiESEL was rather low (29–34, 41–43), despite the OMD recommendation being exceeded, particularly by the older children. Although meat is an important dietary source of iron, median iron intakes in KiESEL were below the specific reference values, particularly in toddlers (36). A higher meat consumption to increase iron intake does not seem advisable, as in adulthood, it was associated with higher all-cause mortality, cardiovascular and cancer mortality (44) as well as a higher risk for T2DM (45). A higher consumption of legumes, whole grains, and certain vegetables during childhood could reduce cardiovascular disease risk through its high fiber content (46, 47), while increasing iron uptake in case good bioavailability is ensured. However, vegetable consumption (including legumes) in KiESEL was below the recommended values in nearly all toddlers and preschoolers. Among the various age groups, vegetable consumption (per 1,000 kcal) was highest in 1-year-olds and did not differ between ages 2, 3, 4, and 5 years, indicating a relative stability during these years. Similar results were found in a longitudinal study from the United States showing a low but stable vegetable consumption between 3 and 7 years of age (48). Also, other European countries report low vegetable consumption, similar to KiESEL (29, 33, 34, 41). Together, these data suggest that interventions to increase long-term vegetable consumption are needed right at the start of complementary feeding.

KiESEL preschoolers did not meet the OMD recommendations for **milk and milk products**. In a European comparison, dairy consumption in KiESEL was on the lower side (29–32, 34, 41–43). Still, milk and milk products were the main dietary source for calcium in KiESEL (own unpublished data). Consistent with the observed below-reference consumption of milk and milk products in preschoolers, calcium intake was below the dietary reference value in this age group (36). Some authors argue that calcium requirements can also be covered by the consumption of other foods, such as broccoli, tofu, nuts, and beans (49). However, consumption of such plant-based calcium-rich foods was low in KiESEL. The available evidence suggests that consumption of cow’s milk might reduce the incidence of dental caries, support dental health, and improve bone health in primary-school children (50). While the exact mechanisms for these effects are unclear, calcium, required for tooth and bone formation, might play a role (50). To meet the current dietary reference value for calcium, preschoolers either need to increase consumption of milk and milk products or of other calcium sources.

The observed disbalances in early diet might mark the starting point of an unfavorable development, as studies show that dietary patterns seem to be stable throughout the early years of life: Similar to our finding that features of an unhealthy dietary pattern already emerge at age 2 years, children following a rather unhealthy dietary pattern when 2 years old were more likely to still consume the pattern 1 and 3 years later (51). Overall, diet quality appears to remain stable from age 3 years until age 7 years, even though consumption of single food groups changed over time (48). As KiESEL toddlers had a median age of 2.5 years, most children in our sample were already at an age where dietary patterns are likely to become stable. Generally, young children’s dietary patterns are influenced by parental diet and educational level as well as family income (52). However, changing such early programmed food preferences requires efforts not only from caregivers but also through measures at the policy level, such as banning marketing for unhealthy foods targeted at children (8).

In addition, other influencing environmental factors need to be considered, such as the COVID-19-pandemic that might have worsened diet quality in young children. Regional German studies suggested that weight gain exceeded physiological levels in toddlers and preschoolers during the pandemic (53), similar to findings of international studies (54). However, at least during the first lockdown, dietary data of German children (3–18 years) with high SES showed no changes in the consumption of unfavorable foods, fruit and vegetables (55). Further nationwide dietary surveys in this age group are needed to compare the post-pandemic food consumption of toddlers and preschoolers living in Germany with that observed in KIESEL.

Major strengths of this study are its detailed data on food consumption collected with weighed food records [considered the gold standard among dietary assessment methods (56)], the representative sampling process, and the application of a sample weighting factor in the statistical analyses. It should be noted though that the proportion of families with low SES in the sample was low compared to the reference population [KiGGS (17)], limiting generalizability. Importantly, as a low SES is known to be associated with lower diet quality (52), the presented results might overestimate diet quality of children living in Germany. Due to the high respondent burden regarding the diet assessment method used, some reactivity bias is likely (56). The low consumption of eggs as well as fats and oils is probably due to not disaggregating all complex foods into their individual components (e.g., cakes). Consequently, the real consumption is expected to be higher. Further, we conducted an exploratory study without defining *a priori* hypotheses. Multiple test adjustment was only performed within each food group, but not for the total number of tests. As only a few children of our sample were classified as having overweight or obesity, most likely related to the overall high or medium SES background in the sample, generalizability of the results of the subgroup analysis is limited. Data on physical activity were not available for all age groups in KiESEL. As measure for comparing age groups, g per 1,000 kcal was chosen to factor in age-specific differences in energy intake. Lastly, the presented data along the early life axis were cross-sectional, and thus, future validation studies based on longitudinal data are required. Nevertheless, the detailed analysis of multiple age groups between ages 1 and 5 years is another strength, giving valuable insights concerning the most relevant phase for interventions addressing the formation of healthy diet patterns.

In conclusion, the identified dietary disbalances—i.e., excess consumption of unfavorable foods, together with low vegetable and high meat consumption—at a very young age gives cause for concern, particularly as it appears to emerge as early as age 2 years. The vulnerable early phase of life requires more age-specific and in-depth analyses of longitudinal datasets to identify possible turning points, potentially leading to more age-specific FBDGs. Sex-specific differences, especially the higher consumption of unfavorable foods in preschool boys, have to be considered. To change the nutritional environment of young children, influencing factors such as parental diet quality and the family’s socioeconomic background need to be analyzed precisely, thereby facilitating joint efforts on multiple levels including policy involvement.

## Data availability statement

The data analyzed in this study is subject to the following licenses/restrictions: Data described in the manuscript, code book, and analytic code will be made available upon request pending application and approval. Requests to access these datasets should be directed to TH, thorsten.heuer@mri.bund.de.

## Ethics statement

The studies involving humans were approved by the Berlin Chamber of Physicians (Eth-28/13). The studies were conducted in accordance with the local legislation and institutional requirements. Written informed consent for participation in this study was provided by the participants’ legal guardians/next of kin.

## Author contributions

CS: Conceptualization, Formal analysis, Methodology, Writing – original draft. SJ: Conceptualization, Formal analysis, Methodology, Writing – original draft. LB: Conceptualization, Methodology, Writing – review & editing. FW: Methodology, Writing – review & editing. A-KB: Methodology, Writing – review & editing. ASc: Methodology, Writing – review & editing. TH: Conceptualization, Methodology, Project administration, Writing – review & editing. ASt: Conceptualization, Methodology, Project administration, Writing – review & editing. SR: Writing – review & editing. SSGB: Methodology, Writing – review & editing. RE: Conceptualization, Methodology, Supervision, Writing – review & editing.
