# Supplementary material for: Unfavorable food consumption in children up to school entry age: results from the nationwide German KiESEL study
Source: Front Nutr. 2024 Jul 1;11:1335934. doi: 10.3389/fnut.2024.1335934 (PMC11246898; doi:10.3389/fnut.2024.1335934)
Supplement: Supplementary file 1 [file Data_Sheet_1.pdf]

Supplementary Material

1 Supplementary Tables

Table S1: Reporting checklist for observational studies in nutritional epidemiology – based on the STROBE-nut guidelines (1)

		Reporting Item	*Page Number*
**Title and abstract**			
Title	#1a	Indicate the study's design with a commonly used term in the title or the abstract	p. 1
Abstract	#1b	Provide in the abstract an informative and balanced summary of what was done and what was found	p. 1
	#nut-1	State the dietary/nutritional assessment method(s) used in the title or in the abstract.	p. 1
**Introduction**			
Background / rationale	#2	Explain the scientific background and rationale for the investigation being reported	p. 2
Objectives	#3	State specific objectives, including any prespecified hypotheses	p. 2
**Methods**			
Study design	#4	Present key elements of study design early in the paper	p. 2–3
Setting	#5	Describe the setting, locations, and relevant dates, including periods of recruitment, exposure, follow-up, and data collection	p. 2–3
	#nut-5	Describe any characteristics of the study settings that might affect the dietary intake or nutritional status of the participants, if applicable.	Dietary assessment: p. 2–3, SES and region: Table 1, weighting factor: p. 4
Eligibility	#6a	Cohort study: Give the eligibility criteria and the sources and methods of selection of participants. Describe methods of follow-up. Case-control study: Give the eligibility criteria and the sources and methods of case ascertainment and control selection. Give the rationale for the choice of cases and controls. Cross-sectional study: Give the eligibility criteria, and the sources and methods of selection of participants.	n/a, sampling performed within KiGGS Wave 2 (2)
	#6b	Cohort study: For matched studies, give matching criteria and number of exposed and unexposed. Case-control study: For matched studies, give matching criteria and the number of controls per case.	n/a, cross-sectional study
	#nut-6	Report any particular dietary, physiologic, or nutritional characteristics that were considered when selecting the target population.	n/a, representative sample

		Reporting Item	*Page Number*
Variables	#7	Clearly define all outcomes, exposures, predictors, potential confounders, and effect modifiers. Give diagnostic criteria, if applicable	n/a, exploratory dietary survey
	#nut-7.1	Clearly define foods, food groups, nutrients, or other food components (e.g., preparation method, taxonomical descriptors, classification, chemical form).	p. 3, Table S2
	#nut-7.2	When calculating dietary patterns, describe the methods to obtain them and their nutritional properties.	n/a, no dietary patterns calculated
Data sources and measurement	#8	For each variable of interest give sources of data and details of methods of assessment (measurement). Describe comparability of assessment methods if there is more than one group. Give information separately for exposed and unexposed groups if applicable.	p. 2–3
	#nut-8.1	Describe the dietary assessment method(s) (e.g., portion size estimation, number of days and items recorded, how it was developed and administered, and how quality was ensured); report if and how supplement intake was assessed.	p. 2–3
	#nut-8.2	Describe and justify food-composition data used; explain the procedure to match food composition with consumption data; describe the use of conversion factors, if applicable	n/a, no food composition database used
	#nut-8.3	Describe the nutrient requirements, recommendations, or dietary guidelines and the evaluation approach used to compare intake with the dietary reference values, if applicable	p. 3
	#nut-8.4	When using nutritional biomarkers, additionally use the STROBE-ME; report the type of biomarkers used and usefulness as dietary exposure markers	n/a, no biomarkers used
	#nut-8.5	Describe the assessment of nondietary data (e.g., nutritional status and influencing factors) and timing of the assessment of these variables in relation to dietary assessment	p. 3
	#nut-8.6	Report on the validity of the dietary or nutritional assessment methods and any internal or external validation used in the study, if applicable	p. 3, 8
Bias	#9	Describe any efforts to address potential sources of bias	weighting factor: p. 4, misreporting: p. 3–4
	#nut-9	Report how bias in dietary or nutritional assessment was addressed (e.g., misreporting, changes in habits as a result of being measured, data imputation from other sources).	p. 3–4
Study size	#10	Explain how the study size was arrived at	n/a, sampling performed within KiGGS Wave 2 (2)
Quantitative variables	#11	Explain how quantitative variables were handled in the analyses. If applicable, describe which groupings were chosen, and why	p. 4;<br>age groups: p. 2

		Reporting Item	*Page Number*
	#nut-11	Explain categorization of dietary/nutritional data (e.g., use of N-tiles and handling of nonconsumers) and the choice of reference category, if applicable.	p. 4, food groups: p. 3
Statistical methods	#12a	Describe all statistical methods, including those used to control for confounding	p. 4
Subgroups and interactions	#12b	Describe any methods used to examine subgroups and interactions	p. 4
Missing data	#12c	Explain how missing data were addressed	p. 2 (missing food records); no missing food consumption data of included participants
Loss to follow up	#12d	Cohort study: if applicable, explain how loss to follow-up was addressed. Case-control study: if applicable, explain how matching of cases and controls was addressed. Cross-sectional study: if applicable, describe analytical methods taking account of sampling strategy.	n/a, no such methods used
Sensitivity analysis	#12e	Describe any sensitivity analyses	n/a, no sensitivity analyses performed
	#nut-12.1	Describe any statistical method used to combine dietary or nutritional data, if applicable.	n/a, no data combined
	#nut-12.2	Describe and justify the method for energy adjustments, intake modeling, and use of weighting factors, if applicable	p. 4
	#nut-12.3	Report any adjustments for measurement error (i.e., from a validity or calibration study).	n/a, no adjustments performed
**Results**			
Participants	#13a	Report numbers of individuals at each stage of study—eg numbers potentially eligible, examined for eligibility, confirmed eligible, included in the study, completing follow-up, and analysed. Give information separately for exposed and unexposed groups if applicable.	p. 2, Figure S1
Non-participation	#13b	Give reasons for non-participation at each stage	n/a, refer to Nowak et al., 2022 (3)
Participant journey	#13c	Consider the use of a flow diagram	Figure S1
	#nut-13	Report the number of individuals excluded on the basis of missing, incomplete, or implausible dietary and nutritional data.	p. 2, Figure S1
Descriptive data	#14a	Give characteristics of study participants (eg demographic, clinical, social) and information on exposures and potential confounders. Give information separately for exposed and unexposed groups if applicable.	p. 4, Table 1
Missing data	#14b	Indicate number of participants with missing data for each variable of interest	Table 1, else no missing data
Follow-up time	#14c	Cohort study: Summarise follow-up time (eg, average and total amount)	n/a, cross-sectional study
	#nut-14	Give the distribution of participant characteristics across the exposure variables, if applicable; specify if food	no exposure variables; food consumption for total population: p. 5

		Reporting Item	*Page Number*
		consumption for the total population or consumers only was used to obtain results	
Outcome data	#15	Cohort study: report numbers of outcome events or summary measures over time. Case-control study: report numbers in each exposure category, or summary measures of exposure. Cross-sectional study: report numbers of outcome events or summary measures.	summary measures: p. 4–5, Tables 1–2, Tables S4–S9
Main results	#16a	Give unadjusted estimates and, if applicable, confounder-adjusted estimates and their precision (eg, 95% confidence interval). Make clear which confounders were adjusted for and why they were included	Table 2, Tables S4–S9 (no adjustments)
Category boundaries	#16b	Report category boundaries when continuous variables were categorized	p. 2
Relative and absolute risks	#16c	If relevant, consider translating estimates of relative risk into absolute risk for a meaningful time period	n/a, no RR calculated
	#nut-16	Specify if nutrient intakes are reported with or without the inclusion of dietary supplement intake, if applicable.	n/a, no nutrient intake calculated
Other analyses	#17	Report other analyses done—eg analyses of subgroups and interactions, and sensitivity analyses	Subgroup analyses: Toddlers/preschoolers: p. 5, Tables 2, S4–S6, Figure 1 Overweight/obesity: p. 5–6, Table S7 Year by year: p. 6, Tables S8, S9, Figure 2
	#nut-17	Report any sensitivity analysis (e.g., exclusion of misreporters or outliers) and data imputation, if applicable	n/a, not performed
**Discussion**			
Key results	#18	Summarise key results with reference to study objectives	p. 7
Limitations	#19	Discuss limitations of the study, taking into account sources of potential bias or imprecision. Discuss both direction and magnitude of any potential bias.	p. 8
	#nut-19	Describe the main limitations of the data sources and assessment methods used and implications for the interpretation of the findings	p. 8
Interpretation	#20	Give a cautious overall interpretation considering objectives, limitations, multiplicity of analyses, results from similar studies, and other relevant evidence.	p. 7–8
	#nut-20	Report the nutritional relevance of the findings, given the complexity of diet or nutrition as an exposure.	p. 8
Generalisability	#21	Discuss the generalisability (external validity) of the study results	p. 8
**Other Information**			
Funding	#22	Give the source of funding and the role of the funders for the present study and, if applicable, for the original study on which the present article is based	p. 9

		Reporting Item	*Page Number*
Ethics	#nut-22.1	Describe the procedure for consent and study approval from ethics committee(s).	p. 2
Data statement	#nut-22.2	Provide data collection tools and data as online material or explain how they can be accessed	p. 9

Table S2: Food groups and OMD recommendations for children aged 1–3 years and 4–6 years[1]

**Food groups covered by OMD (4) (n = 11)**	**Examples**	**Recommended amount (4)**		**Recommended share (4)**
		**1–3 years**	**4–6 years**	**all age groups**
**Eat plenty (plant-based foods)**				
Beverages	Water, tea, malt coffee, commercial[2] teas and juices (ready-to-drink products)	600 mL/day	750 mL/day	37 %TFC
Vegetables	Vegetables, legumes, soy and pure soy products (i.e. unflavoured tofu), CCFs with vegetables as main ingredients	190 g/day	230 g/day	12 %TFC
Fruit	Fruit, fruit juices, smoothies, CCFs with fruit as main ingredient	180 g/day	210 g/day	11 %TFC
Carbohydrate foods[3]:				13 %TFC
Bread and cereals	Bread and bread rolls, cereals, cornflakes and muesli (mainly plain cereal flakes), CCFs with cereals as main ingredients	110 g/day	130 g/day	7 %TFC
Starchy side dishes	Rice, pasta, potatoes and potato products	100 g/day	120 g/day	6 %TFC
**Eat in moderation (animal-based foods)**				
Milk and milk products[4]	Milk and milk products (non-sweetened), cheese, curd, human milk, milk-based drinks intended for young children, infant and follow-on formula (ready-to-drink products), CCFs with milk as main ingredients	300 g/day	350 g/day	18 %TFC
Meat and sausages	Meat, game, poultry, sausages	30 g/day	35 g/day	2 %TFC
Fish[5]	Fish, crustaceans, shellfish, fish products	9 g/day	10 g/day	1 %TFC
Eggs[6]	Eggs	13 g/day	17 g/day	1 %TFC
**Eat sparingly**				
Fats and oils	Vegetable oils, margarines, butter, lard	20 g/day	20 g/day	1 %TFC
**Tolerated**				
Unfavorable foods	Sweets, sweet spreads, ice cream, desserts, cakes, cookies, pancakes, sweetened breakfast cereals, sweetened milk-products (e.g. sweetened fruit yoghurt), salty snack products (e.g. crisps, salted nuts), soft drinks, nectars and mixed products, coffee, cocoa, meat substitutes (excl. pure tofu), plant-based milk alternatives (PBMAs), ready-made sauces (e.g. ketchup), CCFs with unfavorable foods as main ingredients, sugar added to foods/drinks	max. 115 kcal/day	max. 135 kcal/day	max. 10 E%
**Food group not covered by OMD (n = 1)**				
Nuts[7]	Unsweetened, unsalted nuts, kernels, seeds and nut butters	-	-	-

[1] CCF, commercial complementary foods; E%, percentage of energy intake; OMD, Optimized Mixed Diet; %TFC, percentage of total food consumption
[2] Defined as products specifically labelled for infants and young children
[3] For this analysis, the two groups of 'bread and cereals' and 'starchy side dishes' were combined into the group of 'carbohydrate foods', as these serve as mutual substitutes.
[4] Recommendation given in milk equivalents.
[5] Calculated from the weekly recommendation (1/7 of the weekly recommendation)
[6] Calculated from the weekly recommendation (1/7 of the weekly recommendation), assuming an average weight of an egg of 60 g
[7] No separate food group in the OMD, hence no recommendation available (-).

Table S3: Calculated cut-off values[1] for the classification of under- and over-reporting of energy intake at individual level (5)

		Under-reporting	Over-reporting
	**Age**		
**Cut-off values**	Toddlers (1–3 years)	< 0.92	> 2.12
	Preschoolers (4–5 years)	< 1.06	> 2.42

[1] For the ratio of reported energy intake to estimated basal metabolic rate, based on a physical activity level of 1.4 and 1.6 for children aged 1–3 years and 4–5 years, respectively.

Table S4: Energy intake and food consumption in KiESEL toddlers and preschoolers (in kcal/day or g/day), stratified by sex (median, P5, P95)[1]

	Toddlers (1–3 years)				Preschoolers (4–5 years)			
	Boys n = 254		Girls n = 247		Boys n = 200		Girls n = 189	
	**Median**	**P5, P95**	**Median**	**P5, P95**	**Median**	**P5, P95**	**Median**	**P5, P95**
**Total energy intake (kcal/day)**	1039	705, 1410	945	595, 1375	1349	1063, 1785	1215	873, 1572
**Food groups (g/day)**								
Beverages	370	65, 843	368	55, 935	525	50, 1095	530	217, 1039
Vegetables	56	10, 157	57	12, 220	66	14, 186	72	10, 169
Fruit (total)[2]	185	37, 401	184	13, 435	220	25, 511	207	0, 536
Fruit juice and smoothies	34	0, 227	39	0, 300	72	0, 303	54	0, 361
Nuts	0	0, 4	0	0, 7	0	0, 4	0	0, 6
Carbohydrate foods (total)	142	56, 267	127	59, 252	160	98, 298	180	103, 303
Bread and cereals	74	20, 164	68	15, 150	81	36, 153	83	35, 149
Starchy side dishes	69	12, 139	61	8, 138	77	28, 214	90	24, 189
Milk and milk products[3]	219	21, 549	209	23, 520	201	25, 525	187	55, 423
Meat and sausages	29	0, 87	29	0, 86	52	6, 132	46	10, 117
Eggs	3	0, 26	2	0, 30	5	0, 38	2	0, 41
Fish	0	0, 31	0	0, 30	0	0, 38	0	0, 48
Fats and oils	6	1, 18	5	0, 17	9	1, 22	8	1, 19
Unfavorable foods (total)[2]	143	24, 551	135	24, 350	273	101, 737	173	81, 607
Sweets	61	11, 159	50	10, 144	102	35, 233	86	24, 177
Soft drinks	0	0, 370	0	0, 233	50	0, 516	25	0, 330
Sweetened milk products	30	0, 135	25	0, 127	27	0, 175	28	0, 155
**Combined food groups (g/day)**								
Beverages + soft drinks + juices + smoothies	490	179, 1125	477	160, 1012	733	330, 1283	694	335, 1392
Milk and milk products + sweetened milk products[4]	263	43, 577	257	61, 536	295	47, 683	256	83, 521

[1] Weighted data (n unweighted). KiESEL, Children's Nutrition Survey to Record Food Consumption; P, Percentile
[2] Note that only selected subgroups are presented. Hence, the sum of subgroups does not correspond to the amount of the superordinate group.
[3] As milk equivalents
[4] With sweetened milk products calculated as milk equivalents.

Table S5: Food consumption in KiESEL toddlers (in %TFC or E%), stratified by sex[1]

	**Toddlers (1–3 years)**							
	**Boys** n = 254				**Girls** n = 247			
	**Mean (SD)**	**95% CI (mean)**	**Median**	**P5, P95**	**Mean (SD)**	**95% CI (mean)**	**Median**	**P5, P95**
Beverages (%TFC)	29.6 (13.7)	28.0–31.3	30.0	7.5, 50.0	31.3 (14.8)*	29.4–33.1	30.9	7.6, 58.8
Vegetables (%TFC)	5.5 (4.5)	5.0–6.1	4.4	0.9, 15.8	5.6 (3.9)	5.1–6.0	4.5	1.2, 14.0
Fruit (total, %TFC)	15.5 (8.5)	14.5–16.6	14.3	2.9, 29.2	15.6 (9.2)*	14.4–16.7	15.0	1.0, 33.6
Carbohydrate foods (total, %TFC)	12.0 (5.4)	11.4–12.7	11.7	4.2, 21.9	11.8 (6.3)	11.0–12.6	10.3	5.1, 21.5
Milk and milk products (%TFC)[2]	17.9 (11.5)*	16.5–19.3	16.1	1.6, 39.8	18.6 (11.5)*	17.2–20.1	19.6	1.5, 37.9
Meat and sausages (%TFC)	2.6 (2.0)*	2.4–2.8	2.3	0.0, 6.4	2.8 (2.5)	2.5–3.1	2.6	0.0, 8.6
Eggs (%TFC)	0.6 (0.8)	0.5–0.7	0.2	0.0, 2.1	0.7 (1.0)	0.5–0.8	0.1	0.0, 2.7
Fish (%TFC)	0.5 (0.8)	0.4–0.6	0.0	0.0, 2.1	0.5 (0.9)	0.4–0.6	0.0	0.0, 2.5
Fats and oils (%TFC)	0.5 (0.5)	0.5–0.6	0.4	0.1, 1.4	0.6 (0.5)	0.5–0.6	0.4	0.0, 1.6
Unfavorable foods (total, E%)[3]	27.2 (12.8)*	25.7–28.8	26.5	8.0, 49.8	25.7 (13.4)*	24.1–27.4	24.8	6.6, 50.2
Soft drinks (E%)	2.4 (5.5)	1.7–3.1	0.0	0.0, 13.3	1.8 (3.8)	1.3–2.2	0.0	0.0, 8.1
Sweets (E%)	18.2 (9.6)*	17.0–19.3	18.1	4.4, 37.9	17.5 (10.2)*	16.2–18.7	16.2	2.6, 34.3
Sweetened milk products (E%)	4.4 (5.9)	3.7–5.1	2.8	0.0, 14.0	4.2 (5.2)	3.5–4.8	2.5	0.0, 16.4
**Combined food groups**								
Beverages + soft drinks + juices + smoothies (%TCF)	39.7 (13.9)*	38.0–41.4	41.3	17.6, 64.4	40.6 (15.5)*	38.7–42.5	39.9	15.9, 67.1
Milk and milk products + sweetened milk products (%TFC)[4]	22.0 (11.5)*	20.6–23.4	21.4	3.8, 42.4	22.4 (11.5)*	21.0–23.9	23.2	5.2, 39.4

[1] Weighted data (n unweighted). CI, Confidence interval; E%, percentage of energy intake; KiESEL, Children's Nutrition Survey to Record Food Consumption; P, Percentile; SD, standard deviation; %TFC, percentage of total food consumption

[2] As milk equivalents

[3] Note that only selected subgroups are presented. Hence, the sum of subgroups does not correspond to the amount of the superordinate group.

[4] With sweetened milk products calculated as milk equivalents.

* Significant difference between preschoolers and toddlers of the same sex (see Table S6); **bold** = significant difference between boys and girls within one age group (non-overlapping 95% CIs)

Table S6: Food consumption in KiESEL preschoolers (in %TFC or E%), stratified by sex[1]

	Preschoolers (4–5 years)							
	Boys n = 200				Girls n = 189			
	**Mean (SD)**	**95% CI (mean)**	**Median**	**P5, P95**	**Mean (SD)**	**95% CI (mean)**	**Median**	**P5, P95**
Beverages (%TFC)	**31.6** (15.3)	29.5–33.7	30.5	4.3, 55.3	**36.2** (14.1)*	34.2–38.2	34.5	16.7, 56.7
Vegetables (%TFC)	4.6 (3.2)	4.2–5.0	4.2	0.8, 10.6	4.9 (3.0)	4.5–5.3	4.4	0.6, 11.1
Fruit (total, %TFC)	14.0 (9.0)	12.8–15.2	13.9	1.8, 28.2	13.1 (9.2)*	11.8–14.3	11.9	0.0, 29.4
Carbohydrate foods (total, %TFC)	**10.9** (4.0)	10.3–11.4	9.7	5.9, 19.3	**12.2** (4.8)	11.5–12.8	11.5	6.2, 21.0
Milk and milk products (%TFC)[2]	14.1 (9.4)*	12.8–15.4	12.0	1.2, 30.3	13.3 (7.8)*	12.2–14.3	11.9	3.4, 28.7
Meat and sausages (%TFC)	3.5 (2.6)*	3.2–3.9	3.2	0.4, 9.3	3.4 (2.3)	3.0–3.7	2.9	0.5, 8.0
Eggs (%TFC)	0.6 (0.8)	0.5–0.7	0.3	0.0, 2.3	0.7 (1.1)	0.6–0.9	0.2	0.0, 3.8
Fish (%TFC)	0.5 (1.0)	0.3–0.6	0.0	0.0, 2.4	0.6 (1.1)	0.5–0.8	0.0	0.0, 3.2
Fats and oils (%TFC)	0.6 (0.5)	0.6–0.7	0.5	0.1, 1.5	0.6 (0.5)	0.5–0.7	0.5	0.1, 1.4
Unfavorable foods (total, E%)[3]	**35.9** (11.6)*	34.4–37.5	35.8	17.9, 56.0	**31.7** (10.7)*	30.2–33.2	29.9	17.2, 49.7
Soft drinks (E%)	3.3 (5.0)	2.6–3.9	1.5	0.0, 17.3	2.1 (3.7)	1.6–2.6	0.8	0.0, 7.7
Sweets (E%)	24.5 (11.4)*	23.0–26.1	23.4	8.5, 47.0	21.8 (10.0)*	20.4–23.2	20.5	6.9, 38.2
Sweetened milk products (E%)	4.6 (7.1)	3.6–5.6	2.3	0.0, 15.9	4.3 (5.4)	3.6–5.1	2.6	0.0, 14.4
**Combined food groups**								
Beverages + soft drinks + juices + smoothies (%TCF)	46.0 (12.3)*	44.4–47.7	45.1	24.6, 68.5	46.7 (11.5)*	45.0–48.3	46.9	30.8, 68.5
Milk and milk products + sweetened milk products (%TFC)[4]	18.7 (10.8)*	17.2–20.1	18.5	3.4, 40.0	17.3 (8.6)*	16.1–18.5	16.1	5.0, 34.4

[1] Weighted data (n unweighted). CI, Confidence interval; E%, percentage of energy intake; KiESEL, Children's Nutrition Survey to Record Food Consumption; P, Percentile; SD, standard deviation; %TFC, percentage of total food consumption

[2] As milk equivalents

[3] Note that only selected subgroups are presented. Hence, the sum of subgroups does not correspond to the amount of the superordinate group.

[4] With sweetened milk products calculated as milk equivalents.

* Significant difference between preschoolers and toddlers of the same sex (see Table S5); **bold** = significant difference between boys and girls within one age group (non-overlapping 95% CIs)

Table S7: Energy intake and food consumption in KiESEL toddlers and preschoolers (in kcal/day, g/day, %TFC or E%), classified as having overweight or obesity, stratified by sex[1]

	**Toddlers (1–3 years)**								**Preschoolers (4–5 years)**							
	**Boys with overweight/obesity** n = 14				**Girls with overweight/obesity** n = 15				**Boys with overweight/obesity** n = 19				**Girls with overweight/obesity** n = 24			
	**Mean (SD)**	**95% CI (mean)**	**Median**	**P5, P95**	**Mean (SD)**	**95% CI (mean)**	**Median**	**P5, P95**	**Mean (SD)**	**95% CI (mean)**	**Median**	**P5, P95**	**Mean (SD)**	**95% CI (mean)**	**Median**	**P5, P95**
Total energy intake (kcal/day)	1088 (206)	969–1206	1082	778, 1580	941 (152)	857–1025	867	756, 1249	1542 (276)	1409–1675	1470	1142, 2282	1436 (267)	1324–1549	1440	1045, 1950
Vegetables (g/day)	66 (45)	40–92	50	19, 200	71 (56)	40–101	55	18, 242	73 (49)	50–97	62	8, 186	89 (61)	63–115	78	21, 198
Milk and milk products (g/day)[2]	255 (164)	161–350	205	79, 663	237 (114)	174–300	236	88, 423	345 (248)	225–464	306	1, 858	227 (136)	169–284	204	20, 424
Meat and sausages (g/day)	37 (24)	23–51	38	0, 81	36 (42)	13–59	35	0, 160	72 (46)	50–95	79	6, 164	60 (39)	44–77	54	10, 140
Unfavorable foods (total, g/day)[3]	194 (204)	76–311	120	8, 799	128 (97)	75–182	137	29, 369	308 (242)	192–425	244	3, 1048	263 (137)	205–320	231	91, 493
Vegetables (%TFC)	5.1 (5.4)	1.9–8.2	3.5	2.1, 23.1	5.5 (4.1)	3.2–7.8	4.4	1.2, 17.8	4.1 (2.6)	2.8–5.3	3.5	0.4, 10.2	5.2 (3.7)	3.7–6.8	4.3	1.6, 13.3
Milk and milk products (%TFC)[2]	18.1 (12.5)	10.9–25.3	13.6	5.4, 49.6	18.4 (8.1)	13.9–22.9	18.9	5.9, 32.1	19.4 (14.2)	12.6–26.3	17.1	0.0, 57.1	13.4 (7.8)	10.1–16.7	12.5	2.0, 25.0
Meat and sausages (%TFC)	2.4 (1.5)	1.5–3.2	2.2	0.0, 4.4	2.9 (3.5)	1.0–4.8	2.9	0.0, 13.0	4.0 (2.5)	2.8–5.2	4.1	0.4, 10.2	3.7 (2.6)	2.6–4.8	3.4	0.5, 8.3
Unfavorable foods (total, E%)[3]	25.8 (10.4)	19.7–31.8	24.1	2.2, 44.2	23.0 (13.4)	15.6–30.4	17.6	6.2, 59.2	30.8 (14.3)	23.9–37.7	30.8	1.0, 56.5	30.3 (9.9)	26.1–34.5	29.1	17.2, 44.8

[1] Unweighted data. Overweight and obesity were defined as follows: children ≤ 60 months: BMI z-score > 2 to ≤ 3 SD overweight, > 3 SD obesity; children ≥ 61 months: BMI z-score > 1 to ≤ 2 SD overweight, > 2 SD obesity (6). CI, confidence interval; E%, percentage of energy intake; KiESEL, Children's Nutrition Survey to Record Food Consumption; P, Percentile; SD, standard deviation; %TFC, percentage of total food consumption

[2] As milk equivalents

[3] Note that only selected subgroups are presented. Hence, the sum of subgroups does not correspond to the amount of the superordinate group.

[4] With sweetened milk products calculated as milk equivalents.

Table S8: Food consumption in KiESEL toddlers by year of age (in g/1000 kcal)[1]

	**1 year** n = 190				**2 years** n = 164				**3 years** n = 147			
	**Mean (SD)**	**95% CI (mean)**	**Median**	**P5, P95**	**Mean (SD)**	**95% CI (mean)**	**Median**	**P5, P95**	**Mean (SD)**	**95% CI (mean)**	**Median**	**P5, P95**
Beverages (g/1000 kcal)	399 (293)	355–443	326	62, 1141	451 (355)	397–506	373	40, 1113	404 (217)	372–437	380	120, 797
Vegetables (g/1000 kcal)	85 (61)	76–94	67	9, 222	70 (50)	63–78	66	11, 157	57 (50)	50–65	45	12, 170
Fruit (total, g/1000 kcal)	194 (113)	177–211	181	2, 400	186 (111)	169–203	181	31, 385	202 (125)	183–220	186	51, 468
Nuts (g/1000 kcal)	1 (3)	0–1	0	0, 1	1 (5)	1–2	0	0, 7	1 (4)	0–2	0	0, 7
Carbohydrate foods (total, g/1000 kcal)	155 (106)	139–171	139	51, 295	150 (54)	142–159	143	77, 253	139 (56)	131–147	135	63, 227
Bread and cereals (g/1000 kcal)	87 (103)	71–102	70	9, 201	75 (38)	69–80	71	27, 151	73 (35)	68–78	73	21, 138
Starchy side dishes (g/1000 kcal)	69 (38)	63–74	65	0, 131	76 (39)	70–82	74	23, 140	66 (41)	60–72	62	15, 128
Milk and milk products (g/1000 kcal)[2]	275 (140)	254–296	246	66, 583	220 (168)	194–245	197	2, 577	198 (142)	177–219	180	19, 398
Meat and sausages (g/1000 kcal)	27 (22)	23–30	23	0, 69	34 (26)	30–38	28	0, 89	38 (26)	34–41	39	3, 89
Eggs (g/1000 kcal)	6 (9)	5–7	1	0, 26	8 (11)	6–10	2	0, 31	9 (13)	7–11	6	0, 32
Fish (g/1000 kcal)	4 (8)	3–5	0	0, 20	8 (11)	6–9	0	0, 32	7 (13)	5–9	0	0, 35
Fats and oils (g/1000 kcal)	6 (5)	5–7	5	0, 16	8 (6)	7–8	6	1, 18	7 (6)	6–8	6	1, 16
Unfavorable foods (total, g/1000 kcal)	142 (151)	119–165	101	12, 366	178 (170)	152–204	132	40, 639	204 (170)	179–230	167	45, 478

[1] Weighted data (n unweighted). CI, Confidence interval; KiESEL, Children's Nutrition Survey to Record Food Consumption; P, Percentile; SD, standard deviation
[2] As milk equivalents

Table S9: Food consumption in KiESEL preschoolers by year of age (in g/1000 kcal)[1]

	4 years<br>n = 164				5 years<br>n = 226			
	**Mean (SD)**	**95% CI (mean)**	**Median**	**P5, P95**	**Mean (SD)**	**95% CI (mean)**	**Median**	**P5, P95**
Beverages (g/1000 kcal)	517 (384)	457–576	410	121, 952	421 (243)	391–451	387	79, 801
Vegetables (g/1000 kcal)	65 (40)	59–71	63	12, 120	56 (39)	51–61	48	5, 130
Fruit (total, g/1000 kcal)	192 (113)	174–209	180	25, 403	153 (115)	139–167	140	0, 365
Nuts (g/1000 kcal)	1 (3)	1–1	0	0, 5	1 (2)	0–1	0	0, 3
Carbohydrate foods (total, g/1000 kcal)	142 (52)	134–150	138	75, 254	140 (46)	134–145	131	84, 219
Bread and cereals (g/1000 kcal)	62 (26)	58–66	56	30, 101	70 (29)	66–73	69	26, 118
Starchy side dishes (g/1000 kcal)	81 (46)	73–88	75	19, 185	70 (43)	65–75	64	23, 154
Milk and milk products (g/1000 kcal)[2]	178 (113)	160–195	160	33, 373	164 (101)	151–176	150	20, 327
Meat and sausages (g/1000 kcal)	38 (26)	34–42	35	5, 93	43 (26)	40–46	42	6, 90
Eggs (g/1000 kcal)	7 (10)	6–9	3	0, 32	8 (12)	7–9	3	0, 31
Fish (g/1000 kcal)	6 (11)	5–8	0	0, 39	6 (13)	5–8	0	0, 40
Fats and oils (g/1000 kcal)	6 (4)	6–7	6	0, 13	8 (5)	7–9	7	1, 17
Unfavorable foods (total, g/1000 kcal)	209 (130)	189–229	188	69, 502	204 (136)	187–221	179	65, 474

[1] Weighted data (n unweighted). CI, Confidence interval; KiESEL, Children's Nutrition Survey to Record Food Consumption; P, Percentile; SD, standard deviation

[2] As milk equivalents

2 Supplementary figures

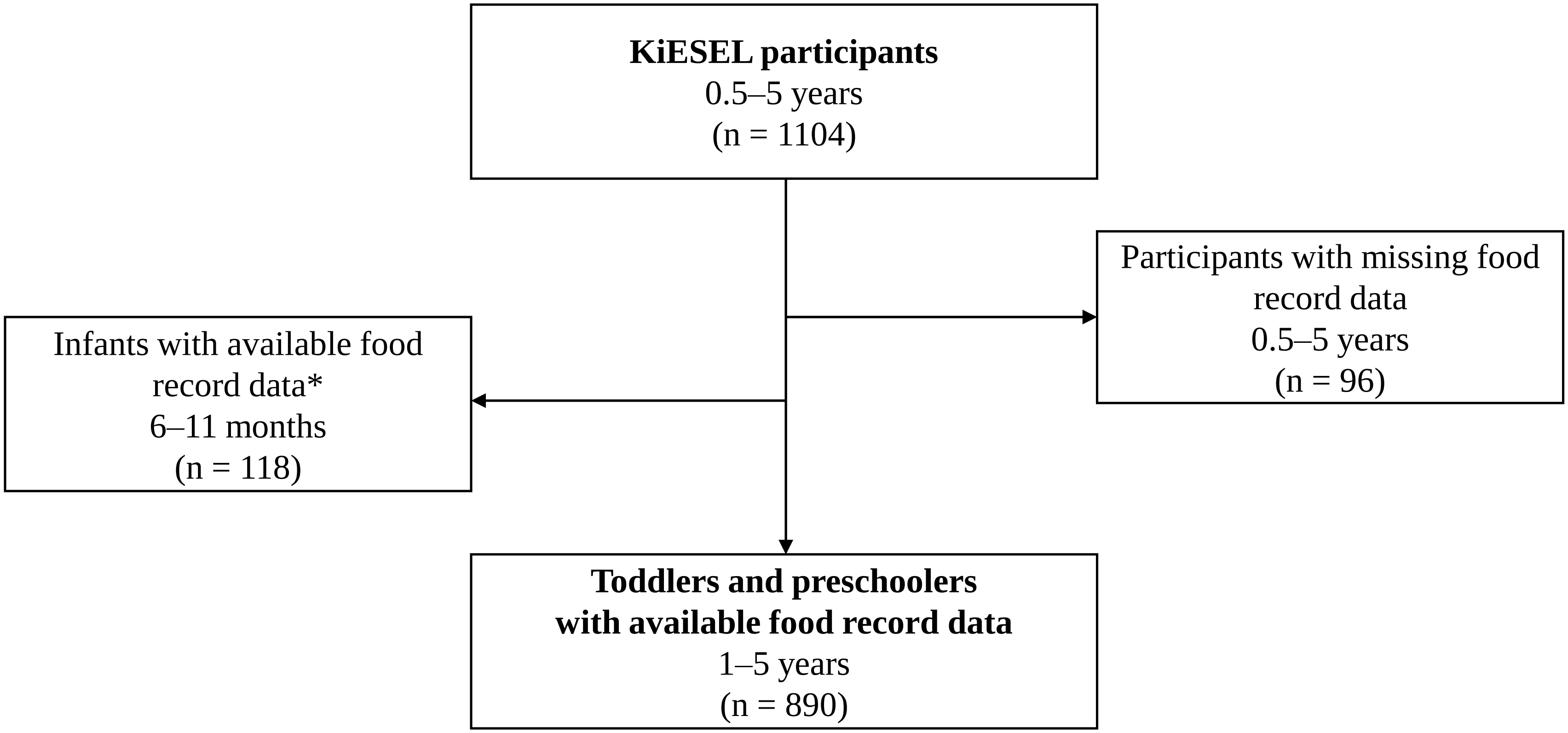

Figure S1: Flow chart of the study population; KiESEL, Children’s Nutrition Survey to Record Food Consumption. * Infants will be reported separately.

$$\text{Milk equivalent factor} = \frac{\text{protein content}_{\text{subgroup 1}}}{\text{protein content}_{\text{raw milk}}}$$

Milk product subgroup	Calculated milk equivalent factor
milk and milk products[2]	1.0
hard cheese	7.0
soft cheese	4.0
sweetened milk products[3]	1.3
human milk	0.3
infant and follow-on formula including milk-based drinks intended for young children	0.4
CCFs with milk or milk products as main ingredient	0.9

[1] The protein content of the subgroup was calculated as protein intake per amount of food, as observed in the sample.

[2] The group of milk and milk products mainly contained milk.

[3] The group of sweetened milk products mainly contained sweetened yoghurt and curd.

Figure S2: Equation (left) and calculated milk equivalent factors per milk product subgroup (right): CCF, commercial complementary foods, the term ‘commercial’ refers to products specifically labelled for consumption by infants and young children

3 References

1. Lachat C, Hawwash D, Ocké MC, Berg C, Forsum E, Hörnell A, et al. Strengthening the Reporting of Observational Studies in Epidemiology - nutritional epidemiology (STROBE-nut): An extension of the STROBE statement. *Nutr Bull* (2016) 41(3):240–51. Epub 20160814. doi: 10.1111/nbu.12217.
2. Mauz E, Gößwald A, Kamtsiuris P, Hoffmann R, Lange M, von Schenck U, et al. New data for action. Data collection for KiGGS Wave 2 has been completed. *J Health Monit* (2017) 2(Suppl 3):S2–28. doi: 10.17886/RKI-GBE-2017-099.
3. Nowak N, Diouf F, Golsong N, Höpfner T, Lindtner O. KiESEL – the children's nutrition survey to record food consumption for the youngest in Germany. *BMC nutrition* (2022) 8(1):64. doi: 10.1186/s40795-022-00527-6.
4. Kersting M, Kalhoff H, Lücke T. Von Nährstoffen zu Lebensmitteln und Mahlzeiten: das Konzept der Optimierten Mischkost für Kinder und Jugendliche in Deutschland [From Nutrients to Food and Meals: The Concept of the Optimized Mixed Diet for Children and Adolescents in Germany]. *Aktuel Ernahrungsmed* (2017) 42(4):304–15. doi: 10.1055/s-0043-116499.
5. European Food Safety Authority (EFSA). Example of a protocol for identification of misreporting (under- and overreporting of energy intake) based on the PILOT-PANEU project. 2013). Available from: www.efsa.europa.eu/sites/default/files/efsa_rep/blobserver_assets/3944A-8-2-1.pdf [Accessed March 7, 2024].
6. de Onis M, Lobstein T. Defining obesity risk status in the general childhood population: which cut-offs should we use? *Int J Pediatr Obes* (2010) 5(6):458–60. Epub 20100317. doi: 10.3109/17477161003615583.
